# Case Report: Not All Neurological Symptoms Respond Well to Penicillin in Patients With Neurosyphilis

**DOI:** 10.3389/fneur.2021.813829

**Published:** 2022-01-24

**Authors:** Xiaoli Wang, Pengying Mu, Wenjuan Zhang, Yonghong Liu

**Affiliations:** ^1^Department of Neurology, Xijing Hospital, Fourth Military Medical University, Xi'an, China; ^2^Department of Neurology, Xing Yuan hospital of Yulin, Shaanxi, China

**Keywords:** neurosyphilis, penicillin, oral-automatism seizures, rhythmic orofacial involuntary movements, periodic lateralized epileptiform discharges, Argyll Robertson pupil

## Abstract

Patients with neurosyphilis present with a wide range of neurological symptoms, the response of which to penicillin is not well known. In this paper, we analyzed the clinical video-electroencephalogram (EEG) features of neurosyphilis in a 54-year-old man who exhibited with rhythmic orofacial involuntary movements, Argyll Robertson pupil, frequent paroxysmal oral-automatism seizures, periodic lateralized discharges (PLEDs) with triphasic waves, behavioral changes, and memory decline. After treatment with penicillin, PLED and seizures disappeared and behavioral changes and memory decline were significantly improved, but rhythmic orofacial involuntary movements and Argyll Robertson pupil persisted, which indicates an irreversible characteristic of late stage neurosyphilis syndromes.

## Background

Neurosyphilis shows a wide range of clinical symptoms, including seizures, poor vision, stroke, confusion, and personality changes (1). If untreated, the infections may involve the brain parenchyma, and the conditions would be worsened with the appearance of general paresis, tabes dorsalis, and Argyll–Robertson pupil (2). Until now, the response of different clinical symptoms to penicillin is little known. We hereby describe a case of neurosyphilis who presented with severe orofacial involuntary movements, Argyll Robertson pupil, and frequent oral automatism seizures. Meanwhile, his electroencephalogram (EEG) revealed periodic lateralized discharges (PLEDs) consisting of triphasic waves over the right temporal region. After treatment with penicillin, PLED disappeared and seizures diminished, but rhythmic orofacial involuntary movements and Argyll Robertson pupil seem to be unresponsive to treatment and still persisted during wakefulness.

## Case Report

A 54-year-old man presented to our hospital with frequent epileptic seizures. The medical history consisted of a 4- to 5-year behavioral change and memory decline, 3 years of involuntary movements of the face and mouth along with sharp clicking sound, and occasional paroxysmal vertigo and tinnitus. By admission, he has taken on valproate at the dose of 50 mg, twice a day for several months but got no obvious benefit. General physical examination was normal. No cerebellar and sensory deficits were detected, and his gait was quite natural. All the cranial nerves were normal on examination except the Argyll Robertson pupil which showed no reaction to the light (Supplementary Video 1). Brain MRI revealed right temporal lobe atrophy. Serologic tests and CSF analysis were both positive for syphilis and so were the serum rapid plasma regain (titer 1:2) and treponema pallidum particle agglutination (TPPA) assay. The serum toludine red unheated serum test (TRUST) titer was 1:2. Cerebrospinal fluid (CSF) TPPA and fluorescent treponemal antibody absorption (TA-Abs) analysis unveiled similar indication. The WBC count and immunoglobulin G in CSF were 20 × 10^6^/L and 51.6 mg/L, respectively, higher than normal. The protein (36 mg/dl) and glucose (3.28 mmol/L) levels were in regular range.

### EEG Findings at Admission

**Figure 1:**
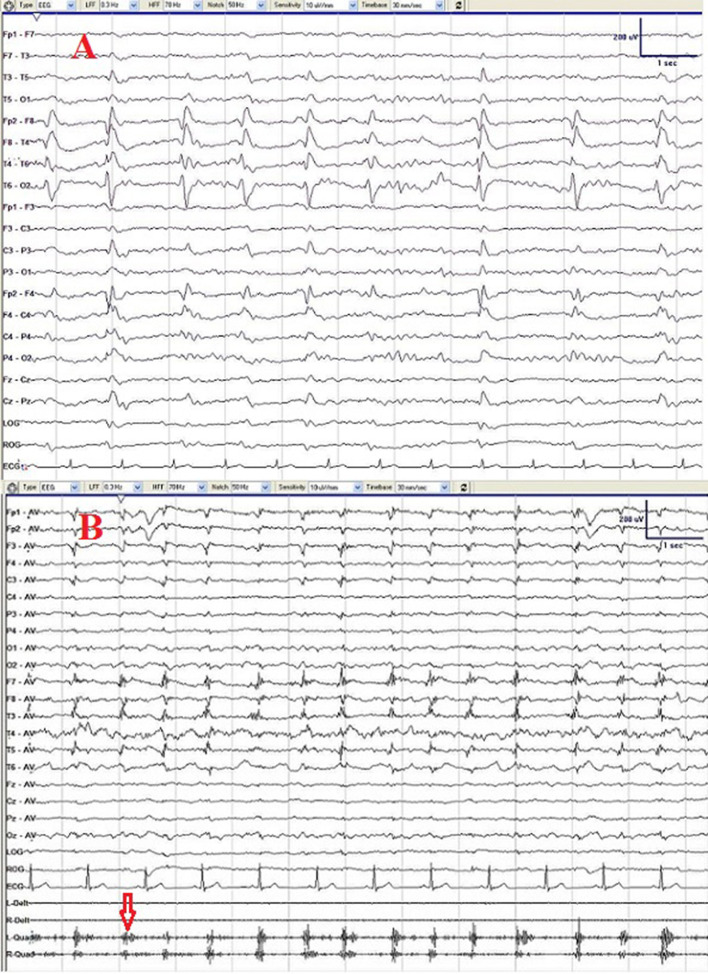
Initial electroencephalogram (EEG) **(A)** showing periodic lateralized discharge (PLED) consisting of triphasic waves over the right temporal region. After treatment with penicillin for 2 courses, PLED disappeared and repeated EEG **(B)** showed only slowing waves and muscle artifacts produced by persisted rhythmic orofacial involuntary movements (red arrow).

Video-EEG monitoring showed PLEDs consisting of triphasic waves over the right temporal region (Figure 1A). In total, 8 times seizures were recorded, which presented as oral and manual automatism and only lasting about 1 min and occurring during non-rapid eye movement (NREM) sleep (Supplementary Video 2). The ictal EEG confirmed a right temporal-frontal region onset theta wave evolution. Meanwhile, rhythmic orofacial involuntary movements arose in wakefulness and disappeared during sleep (Supplementary Video 3), indicating an uncontrolled contraction of masticatory muscles.

### Treatment and Course

The patient was treated with penicillin (24 MU of intravenous aqueous penicillin G daily, divided every 4 h) for 14 days as a course. After two courses of treatment with penicillin plus valproate 50 mg orally two times/day, significant improvement was observed with his seizures and behavioral symptoms. The patient refused to get CSF test at the time of his second visit, only EEG and Mini-Mental State Examination (MMSE) were performed, and the EEG performed three months after the treatment found no PLEDs and only slow waves over the right temporal region. The MMSE of the patient was 22/30 (with the diploma of primary school), implying a normal range of cognition. However, Argyll Robertson pupil and rhythmic orofacial involuntary movements persisted after the treatment even at 7-month follow-up (Figure 1B).

## Discussion

In this study, we reported a case of confirmed neurosyphilis presented with PLEDs in EEG and manifested by frequent seizures, Argyll Robertson pupil, and rhythmic orofacial involuntary movements. After the treatment with penicillin, PLED disappeared and seizures diminished, but rhythmic orofacial involuntary movements and Argyll Robertson pupil still persisted.

Periodic lateralized discharges (PLEDs) are often seen with acute stroke, brain tumors, and inflammatory and infectious diseases (3). Whether PLEDs represent an ictal or interictal phenomena remains highly controversial (4). However, lateralized periodic discharges are considered ictal when related to apparent clinical symptoms (4, 5). The higher WBC count in CSF indicated an inflammatory reaction. The PLEDs in neurosyphilis might be a reflection of enhanced neuronal excitability, which is a treatable condition (6, 7). Moreover, PLEDs in this patient was temporally associated with frequent epileptic seizures and behavioral symptoms. In addition, after treatment with penicillin and anti- seizure medicines, PLEDs disappeared and, similarly, the seizures and behavioral disorders were improved. Therefore, our finding stresses the importance of performing syphilis testing for patients with frequent seizures. We postulated that syphilis induces a subacute state of epileptogenicity as the possible pathology, and the PLEDs may represent the peri-ictal pattern (6, 7).

In our case, the video-EEG captured 8 times of automatism seizures, and the ictal EEG confirmed a right temporal-frontal originated theta wave evolution. This finding agrees with previous demonstrations that the oral automatism seizures are typical focal seizure for medial temporal lobe epilepsy (8), and ictal EEG confirmed right temporal-frontal region onset theta wave evolution. Orofacial involuntary movements are well described in neurosyphilis and have been termed as “the candy sign,” which is considered the clue to the diagnosis of neurosyphilis (9). Although these two kinds of events manifest similarly, involving the facial and masticatory muscles in a stereotyped manner, there are clear differences in electrophysiological feature. First, oral automatism seizures are associated with ictal epileptic discharges, only lasting about 1 min, and occurring during NREM sleep. However, rhythmic orofacial involuntary movements were continuous and persisted during wakefulness without epileptic discharges. Furthermore, after treatment with penicillin, PLED disappeared and seizures diminished, but rhythmic orofacial involuntary movements were unresponsive to treatment and still persisted during wakefulness. Therefore, the neural pathogenesis underlying these two kinds of events would be different.

Patients with frequent seizures may experience psychiatric symptoms during the period around the ictus (10). However, cognitive decline associated with frequent seizures always indicated a potentially reversible condition (10, 11). Memory is probably affected by focal seizures arising from temporal lobe, which is treatable (12). In line with previous findings, there was notable improvement after two courses of treatment with penicillin in our patient (6, 7). The MMSE scored: 22/30 comparable to the diploma of primary school skill. Meanwhile, seizures, PLEDs, and behavioral symptoms were significantly improved by penicillin treatment.

The late stage neurosyphilis with general paresis is characterized by progressive dementia, psychiatric syndromes, personality change, manic delusions, tremor, and dysarthria (1). Pathologically, there is atrophy of the frontal and temporal lobe. The “Lissauer” form of the disease may also affect the cerebellum and basal ganglia (13). Common neurological signs include pupillary abnormalities (Argyll–Robertson pupils), dysarthria, tremors of facial, lingual, and hand muscles (1, 13). Argyll Robertson pupil is pathognomonic of neurosyphilis, particularly, the pathologic lesion is suspected to be in the midbrain that interrupts the pupillary light reflex pathway (14). The mechanism underlying the rhythmic orofacial involuntary movements in neurosyphilis remains unknown, but a wealth of data implicates the basal ganglia may be involved (15–17). Orofacial involuntary movements exhibit with tardive syndromes which presents as vacuous chewing movements accounted by dopamine super sensitivity theory as high expression of D2 receptors has been identified (18). There is some evidence to support the theory. Particularly, an increased number of D2 receptors have been found to correlate with vacuous chewing movement (18). Therefore, the defective dopaminergic pathway in basal ganglia region may also induce the orofacial involuntary movements.

Although neurosyphilis is a treatable condition, the reversibility is dependent on the severity and duration of the disease. Hence, penicillin probably does not improve late neurosyphilitic syndromes (13). Like in our case, Argyll Robertson pupils and rhythmic orofacial involuntary movements did not react to the medical intervention (17).

## Conclusion

Overall, neurosyphilis related neurologic disorders are diverse. The response of which to penicillin varied according to the severity and duration of the disease. Hence, penicillin probably has little effect on late neurosyphilitic syndromes.

## Data Availability Statement

The original contributions presented in the study are included in the article/Supplementary Material, further inquiries can be directed to the corresponding author/s.

## Ethics Statement

The studies involving human participants were reviewed and approved by Xijing Hospital Research Ethics Committee. The patients/participants provided their written informed consent to participate in this study. Written informed consent was obtained from the individual(s) for the publication of any potentially identifiable images or data included in this article.

## Author Contributions

XW and PM conducted the clinical assessment. WZ conducted the video recording. XW, PM, and YL assisted in the video editing, initial draft, and revisions of manuscript. XW and YL assisted in the interpretation of clinical data and the revision of the manuscript.

## Conflict of Interest

The authors declare that the research was conducted in the absence of any commercial or financial relationships that could be construed as a potential conflict of interest.

## Publisher's Note

All claims expressed in this article are solely those of the authors and do not necessarily represent those of their affiliated organizations, or those of the publisher, the editors and the reviewers. Any product that may be evaluated in this article, or claim that may be made by its manufacturer, is not guaranteed or endorsed by the publisher.

## Supplementary Material

Supplementary Video 1 The pupils constricted poorly to light stimulus. There is no apparent change in pupil size.

Supplementary Video 2 The pre-ictal electroencephalogram (EEG) shows a focal abnormal triphasic waves and slow waves over the right temporal region during non-rapid eye movement (NREM) sleep, followed by the ictal EEG, revealing a seizure. Ictal EEG presented with attenuation of background, along with movement artifact and then followed by 3–5 Hz slow waves evolution during the seizure. The ictal semiology was characterized by sudden awakening from sleep with movement artifact, and then followed by oral automatism seizures.

Supplementary Video 3 The rhythmic orofacial involuntary movements detected in the patient is mild, continuous, and repetitive, involving the lower facial and masticatory muscles in a stereotyped manner and producing sharp clicking sound.
